# Supplementary material for: Anti-Inflammatory Action of Heterogeneous Nuclear Ribonucleoprotein A2/B1 in Patients with Autoimmune Endocrine Disorders
Source: J Clin Med. 2019 Dec 19;9(1):9. doi: 10.3390/jcm9010009 (PMC7019344; doi:10.3390/jcm9010009)
Supplement: Supplementary file 1 [file jcm-09-00009-s001.pdf]

Table S1: primer sequences used for RT-PCR analysis.

GENE	PRIMER SEQUENCE
PDL-1 (CD274)	Forward primer 5′ TTGCTGAACGCCCCATACAA 3′<br>Reverse primer 5′ GGAATTGGTGGTGGTGGTCT 3′
TGF-β1	Forward primer 5′ GTGGACATCAACGGGTTCACT 3′<br>Reverse primer 5′ ATGAGAAGCAGGAAAGGCCG 3′
COX2	Forward primer 5′ ATCATTCACCAGGCAAATTGC 3′,<br>Reverse primer 5′ GGCTTCAGCATAAAGCGTTTG 3′
HGF	Forward primer 5′ CTC ACA CCC GCT GGG AGT AC 3′<br>Reverse primer 5′ TCC TTG ACC TTG GAT GCA TTC 3′
IL-2	Forward primer 5′ ACCTCAACTCCTGCCACAATG 3′<br>Reverse primer 5′ TGAGCATCCTGGTGAGTTTGG 3′
IFN-γ	Forward primer 5′ ACTGTCGCCAGCAGCTAAAA 3′<br>Reverse primer 5′ TATTGCAGGCAGGACAACCA 3′
ARG-1	Forward primer 5′ GGGTTGACTGACTGGAGAGC 3′<br>Reverse primer 5′ CGTGGCTGTCCCTTTGAGAA 3′
IL-4	Forward primer 5′ CTTCCTGCTAGCATGTGCC 3′<br>Reverse primer 5′ GTTGTGTTCTTGGAGGCAGC 3′
IL-17A	Forward primer 5′ AACCGATCCACCTCACCTTG 3′<br>Reverse primer 5′ TCTCTTGCTGGATGGGGACA 3′
FOX-P3	Forward primer 5′ GCCTTGCCAAAAATACCCCG 3′<br>Reverse primer 5′ TGGGGAGCTCGGCTG 3′

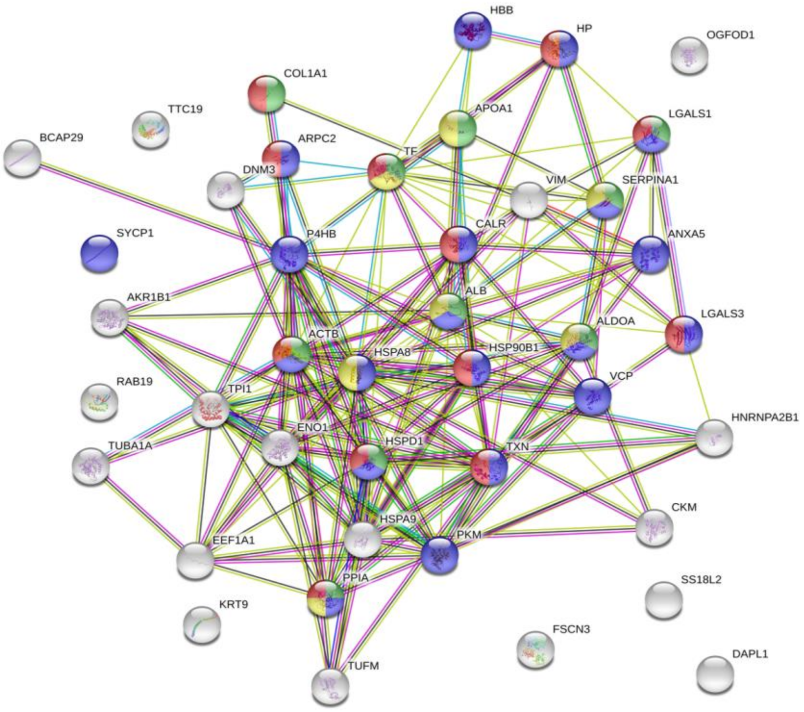

Figure S1: Protein-protein interaction network visualized by STRING database. Interactome derived from the proteins listed in Table S1 using String algorithm. Colours highlight the three top significantly biological processes (response to stress, secretion by cell and immune system process) involved in the protein-protein interrelationships.

Table S2: Proteome and secretome list of the modulated proteins.

ID SPOT	Gene name	Accession number	Protein name	Theoretical Molecular weight (Da)	Theoretical pI	Score	Sequence coverage
C16	ACTB	P60709	Actin, cytoplasmic 1	41737	5,29	130	42%
C34	ACTB	P60709	Actin, cytoplasmic 1	41737	5,29	125	59%
C39	ACTB	P60709	Actin, cytoplasmic 1	41737	5,29	159	52%
C41	AKR1B1	P15121	Aldose reductase	35834	6,52	73	49%
M15	ALB	P02768	Serum albumin	66472	5,67	114	23%
C17	ALDOA	P04075	Fructose-bisphosphate aldolase A	39289	8,39	73	49%
M3	ANXA5	P08758	Annexin A5	35936	4,93	88	41%
M16	APOA1	P02647	Apolipoprotein A-I	28079	5,27	97	40%
C42	ARPC2	O15144	Actin-related protein 2/3 complex subunit 2	34333	6,84	71	21%
C40	BCAP29	Q9UHQ4	B-cell receptor-associated protein 29	28320	9,55	63	19%
C26	CALR a	P27797	Calreticulin	46466	4,29	98	58%
C13	CALR b	P27797	Calreticulin	48200	4,29	79	31%
M5	CKM	P06732	Creatine kinase M-type	43101	6,77	66	23%
M1	COL1A1	P02452	Collagen alpha-1(I) chain	138941	5,6	65	21%
M11	DAPL1	AOPJW8	Death-associated protein-like 1	11879	9,6	60	60%
C3	DNM3	Q9UQ16	Dynamin-3	97746	8,5	66	21%
C8	EEF1A1	P68104	Elongation factor 1-alpha 1	50140	7,1	75	30%
M9	EFTU	P49411	Elongation factor Tu, mitochondrial	49541	7,26	92	22%
M10	ENO1	P06733	Alpha-enolase	47038	6,99	58	11%
M7	ENO1	P06734	Alpha-enolase	47038	6,99	60	12%
M20	ENO1	P06735	Alpha-enolase	47038	6,99	68	11%
C25	ENO1	P06736	Alpha-enolase	47038	6,99	167	44%
C23	ENO1	P06737	Alpha-enolase	47038	6,99	150	45%
M12	FSCN1	Q16658	Fascin	54530	6,84	74	22%
M17	HBB	P68871	Hemoglobin subunit beta	15867	6,81	73	57%
C24	HNRNPA2 B1	P22626	Heterogeneous nuclear ribonucleoproteins A2/B1	37429	8,97	88	18%
C4	HNRNPA2 B1	P22626	Heterogeneous nuclear ribonucleoproteins A2/B1	37429	8,97	64	23%
M8	HNRNPA2 B1	P22627	Heterogeneous nuclear ribonucleoproteins A2/B2	37429	8,97	59	23%
M18	HP	P00738	Haptoglobin	43341	6,13	86	25%
C33	HSCB	Q18IWL3	Iron-sulfur cluster co-chaperone protein HscB, mitochondrial	27422	7,59	63	34%
M4	HSCB	P62937	Peptidyl-prolyl cis-trans isomerase A	18012	7,68	67	42%
C32	HSP90B1	P14625	Endoplasmin	90178	4,73	199	40%
C30	HSP90B1	P14625	Endoplasmin	90178	4,73	66	17%
C28	HSPA8	P11142	Heat shock cognate 71 kDa protein	70767	5,37	86	24%
C27	HSPA8	P11142	Heat shock cognate 71 kDa protein	70767	5,37	89	25%
C29	HSPA9	P38646	Stress-70 protein, mitochondrial	68759	5,44	97	26%
C10	HSPD1	P10809	60 kDa heat shock protein, mitochondrial	57963	5,24	62	25%
C11	HSPD1	P10809	60 kDa heat shock protein, mitochondrial	57963	5,24	99	35%
C1	KRT9	P35527	Keratin, type I cytoskeletal 9	62064	5,14	99	28%

C35	KRT9	P35527	Keratin, type I cytoskeletal 9	62064	5,14	117	26%
C36	KRT9	P35527	Keratin, type I cytoskeletal 9	62064	5,14	84	26%
C38	LGALS1	P09382	Galectin-1	14715	5,3	59	25%
C18	LGALS3	P17931	Galectin-3	26021	8,6	93	49%
C15	P4HB	P07237	Protein disulfide-isomerase	55294	4,69	217	52%
C14	PKM	P14618	Pyruvate kinase PKM	57806	7,95	62	20%
C12	PKM	P14618	Pyruvate kinase PKM	57806	7,95	97	36%
C6	RAB19	A4D1S5	Ras-related protein Rab-19	24399	6,06	55	12%
M2	RIOX2	Q81UF8	Ribosomal oxygenase 2	52800	6,23	66	22%
M13	SERPINA1	P01009	Alpha-1-antitrypsin	44324	5,37	125	38%
M14	SERPINA1	P01009	Alpha-1-antitrypsin	44324	5,37	112	42%
C7	SS18L2	Q9UHA2	SS18-like protein 2	8835	5,57	72	70%
M6	SYCP1	Q15431	Synaptonemal complex protein 1	114191	5,78	64	22%
M19	TF	P02787	Serotransferrin	75195	6,7	88	19%
C19	TPI1	P60174	Triosephosphate isomerase	30791	5,45	67	43%
C20	TPI1	P60174	Triosephosphate isomerase	30791	5,45	141	61%
C37	TTC19	Q6DKK2	Tetratricopeptide repeat protein 19, mitochondrial	42456	5,57	76	13%
C5	TUBA1A	Q71U36	Tubulin alpha-1A chain	50135	4,94	101	36%
C22	TXN	P10599	Thioredoxin	11606	4,82	100	66%
C21	TXN	P10599	Thioredoxin	11606	4,82	124	72%
C31	VCP	P55072	Transitional endoplasmic reticulum ATPase	89191	5,14	241	37%
C2	VIM	P08670	Vimentin	53651	5,05	153	31%
C9	VIM	P08670	Vimentin	53651	5,05		

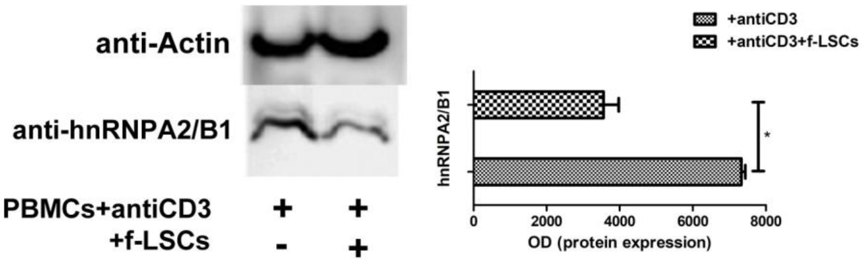

Figure S2: Western blot immunodetection of hnRNPA2/B1 protein. Monodimensional Western blot of hnRNPA2/B1 protein detected by silver stain proteomics is shown (left panel). The respective intensity level of each reactive band in activated PBMCs of AED patients before and after f-LSC treatment is reported in the histogram (right panel).

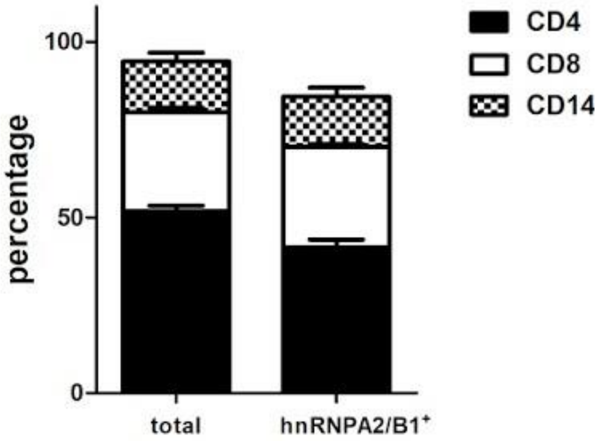

Figure S3. Flow cytometry analysis for hnRNPA2/B1 and CD4+, CD8+ and CD14+subsets in PBMCs of AED patients. Histogram shows the hnRNPA2/B1 expression pattern in fresh CD4+, CD8+ and CD14+ PBMCs collected from AED patients.
